# Students’ understanding of “Women-Centred Care Philosophy” in midwifery care through Continuity of Care (CoC) learning model: a quasi-experimental study

**DOI:** 10.1186/s12912-015-0072-z

**Published:** 2015-04-22

**Authors:** Yanti Yanti, Mora Claramita, Ova Emilia, Mohammad Hakimi

**Affiliations:** Undergraduate Program of Midwifery Education, Estu Utomo Boyolali School of Health Science, Tentara Pelajar Street no. 12, Mudal, Boyolali 57351 Indonesia; Department of Medical Education and Family Medicine Graduate Program, Faculty of Medicine, Gadjah Mada University, Radiopoetro Building 6th floor, Farmako Street no. 1, Sekip Utara, Jogjakarta 55281 Indonesia; Department of Ob-Gyn and Department of Medical Education, Faculty of Medicine, Gadjah Mada University, Farmako Street no. 1, Sekip Utara, Jogjakarta 55281 Indonesia; Department Ob-Gyn Faculty of Medicine, Gadjah Mada University, Jogjakarta, Indonesia

**Keywords:** Midwifery clinical learning, Midwifery care philosophy, Women-centred care, Continuity of care

## Abstract

**Background:**

The philosophy of midwifery education is based on the ‘Women-centred care’ model, which provides holistic care to women. Continuity of care (CoC) is integral to the concept of holistic women-centred care and fundamental to midwifery practice. The objective of this study was to determine any differences in students’ understanding of midwifery care philosophy between students who underwent the CoC learning model and those who underwent the fragmented care learning model.

**Method:**

We used a quasi-experiment design. This study was conducted by all final year midwifery students at two schools of midwifery in Indonesia. Fifty four students from one school attended 6 months of clinical training using the CoC learning model. The control group was comprised of 52 students from the other school. These students used the conventional clinical training model (the fragmented care learning model). The independent T-test using SPSS was used to analyse the differences between the two groups of students in terms of understanding midwifey care philosophy in five aspects (personalized, holistic, partnership, collaborative, and evidence-based care).

**Results:**

There were no significant differences between the groups before interventon. There were significant differences between the two groups after clinical training (p < 0.01). The mean post-clinical score of students using all five aspects of the CoC clinical learning model (15.96) was higher than that of the students in the control group (10.65). The CoC clinical learning model was shown to be a unique learning opportunity for students to understand the philosophy of midwifery. Being aligned with midwifery patients and developing effective relationships with them offered the students a unique view of midwifery practice. This also promoted an increased understanding of the philosophy of women-centred care. Zero maternal mortality rate was found in the experiment group.

**Conclusion:**

The results of this study suggest that clinical trainingwith a CoC learning model is more likely to increase students’ understanding of midwifery care philosophy. This in turn improves the quality ofclinical care, thereby enhancing overall health benefits for women.

**Electronic supplementary material:**

The online version of this article (doi:10.1186/s12912-015-0072-z) contains supplementary material, which is available to authorized users.

## Background

### Women-centred as philosophy of midwifery care

The philosophy of midwifery care centers upon women-centred primary health care services, which rely on the relationships between women and their midwives during all women’s life cycle [1]. Midwives care for women during pregnancy, childbirth and in early parenting years. Midwives educate women in healthy lifestyle choices, especially those that center around the profound processes and precious events in women’s reproductive lives. These events are also seen as inherently important to society as a whole.

The critical importance of “women-centred care” in midwifery practices has been reviewed in the literature [2-7], including how it promotes an environment of shared power and responsibility between a woman and her midwife [2,3]. The concept of “women-centred care” is central to midwifery practices and underpins the philosophy statement of the International Confederation of Midwives and the Australian College of Midwives [6,8]. “Women-centred care” is a term used to describe a philosophy of maternity care that promotes a holistic approach by recognizing each woman’s social, emotional, physical, spiritual and cultural needs The expectations and context are defined by the woman herself [5,8]. The fundamental principles of women-centred care ensures a focus on pregnancy and childbirth as the start of family life, not just as isolated clinical episodes. These motherhood phases take into complete account the meanings and values of each woman [5]. Women-centred carein the clinical setting is safe, supportive, and gentle. It is the philosophical foundation of undergraduate midwifery courses, which in turn promotes the understanding needed by midwifery students to care for women holistically [3,9].

### The importance of developing continuity of care as part of midwifery care philosophy

Continuity of midwifery care means that a woman develops a partnership with a midwife to receive care during pregnancy, labour and the postnatal period [10]. A “continuity of care partnership” between a midwife and a patient is defined as having a ‘professional friend’ [11,12]. In this model of care, midwives have enhanced, high-level opportunities to explore and understand pregnancy, labour and birth and the postnatal period.

The continuity of care model in midwifery care is a way to ensure that women and their babies get the best care from midwives throughout the childbirth continuum [6]. One study found that continuity of midwifery care was associated with lower intervention rates than standard maternity care [13]. The study also mentioned that continuity of midwifery care was associated with less use of obstetric interventions during labour and that there was no maternal death. This is relevant in meeting targets 4 & 5 of the MDGs to reduce maternal and infant mortality.

In a study by Aune et al. [14], continuous care, as opposed to random, fragmented care, enables a close, trusting relationship between a woman and a midwife, and thus it is valuable for both of them. Continuity of care is fundamental to midwifery practice models. It is both a philosophy and a process that enables midwives to provide holistic care and to establish an ongoing partnership with their patients in order to build understanding, support and trust. Continuity of care is facilitated through a one-to-one relationship between the midwife and her patient [5].

Whilst there are many ways in which midwifery care may be organised, midwives can function autonomously as primary care providers. Personalizing (individualizing) care for each woman also includes the ability to refer to other health professionals when necessary [11].

Continuity of care in student midwifery education is defined as the experience of having an ongoing midwifery relationship between students and patients, from initial contact in early pregnancy to the weeks shortly after birth, and across the interface between community and hospital settings. The intention of the continuity of care experience is to enable students to experience continuity with an individual woman through pregnancy, labour, birth and the postnatal period, [15]. It is important for the students to learn the core value of midwifery care philosophy that is Continuity of care (CoC): the fundamental duty of a midwife when delivering women centered care.

### Impacts of the continuity of midwifery care model on student learning

The strength of the CoC experience is the concept of patient-based learning and patient as educator. Students learn directly from the patients, and from other healthcare providers, which then consolidates students’ understanding of women-centred care philosophy. Being actively engaged in the CoC experience assists students in developing and/or confirming a women-centred care philosophy [16]. There is limited research on the nature of the midwifery student relationship with patients and the impact this has on student learning. The Australian College of Midwiveshas proposed “follow-through” experiences in their standards for three-year Bachelor of midwifery education programs; this strategy is a means of ensuring midwifery students have continuity of care experiences [17-19]. A similar model of midwifery student clinical education is known as a “student case-loading” in Bournemouth University [20,21].

Studies that explored the experiences of the first cohort of Bachelor of Midwifery students in Australia noted that students valued the opportunity to develop relationships with women [22] and identified that the follow-through experience provided them with this opportunity [23]. Although these models of care are now more evident, students enjoyed this aspect of their course because of the richness of midwifery experiences encountered. However, recruitment of the participant (patient) is challenging [19].

There are limited studies that specifically discuss students’ understanding of the philosophy of midwifery care. One study’s goal was to gain insight into how continuity of care can increase student understanding of midwifery when the emphasis is on the promotion of normal pregnancy, childbirth and the postnatal period [14].

At present, midwifery students in Indonesia may graduate with a limited knowledge of any model of care, for example, the fragmented midwifery care learning model which is implemented during the clinical placement phase of education. Current Midwifery education in Indonesia consists of a three year program in which knowledge and clinical skills are given simultaneously. Begining in semester three, students undergo a clinical phase which typically a “fragmented care” learning model [24]. To achieve clinical competencies, a student is required to provide a number of midwifery cares, involving different patients, during antenatal, intranatal, and postnatal periods (the fragmented care model).

Conducting research with midwifery students in clinical placements is seen as an ideal setting to explore how students’ experiences influence their understanding of midwifery philosophy of care. The CoC learning model has been proven to provide a more contextual learning experince which is similar to future professional midwife practice [16-20]. However, comprehension of the students about midwifery care philosophy prior to clinical placement has not been measured in any study [16-20]. Therefore, the objective of this study was to examine how the students understand midwifery care philosopy “Women-Centred Care” through the CoC experience as a clinical learning model.

## Methods

A quasi-experimental “the non-equivalent control group pre-post test design” was used in this study. Quasi-experimental studies encompass a broad range of nonrandomized intervention studies. These designs are frequently used when it is not logistically feasible or ethical to conduct a randomized controlled trial. However, in many quasi-experimental studies there may be nonrandom selection of units to both controls and experimentals, and it is not necessarily the same selection variable that governs the selection process for controls and experimentals [25]. The clinical learning model was divided into two locations that involved all students at each institution; therefore, a full experimental design was not possible. Continuity of care was applied to students in the experiment group. Each student followed 2–3 women during pregnancy until postpartum. Students were supported by a midwife tutor linked to their clinical area throughout their clinical experience, and were supervised by a school midwife sign-off mentor. The control group only used the conventional model of fragmented care learning.

In 2014, there were a total of 800 Midwifery schools across Indonesia with total student numbers of approximately 240.000. We selected two Midwifery schools for this study based on similarity of accreditation level, characteristics of curriculum, and number of students. To avoid testing effect, the selected schools were approximately 1500 km (3 hours by plane) apart, thereby decreasing the likelihood that students at both schools would communicate with each other [25]. There was no significant difference in socio-demographic characteristics between the location of the two schools in terms of similarity of languages, tribes, and income. The subjects of this study were all final year midwifery students: 54 students from one school who attended the 6 month clinical placement using a CoC learning model and 52 students from the other school as the control group who underwent a fragmented care learning model. A nonrandomized selection (based on socio-demographic characteristics) was used to determine the sample size at two location that it represented general populations of midwifery students accross Indonesia.

This CoC learning model was delivered to the participants in the experiment group using the CoC learning module (see Additional file 1). This module was produced by the author and involved all parties (students, clinical midwives, midwife teachers and delegations from local midwifery association) [26].

The pre and post-survey questionnaires were comprised of five sections (see Table 1), administered to the students in both groups before and after the study. The first five sections contained questions related to the students’ understanding of the midwifery care philosopy during the clinical experience: (1) personalized care, (2) holistic care, (3) partnership care, (4) collaborative care, and (5) evidence based care. The data reported in this article related to the students’ understanding of midwifery care philosopy “Women-Centred Care” experienced during the clinical placement.

**Table 1:** **Materials of questions and topics asked for students’ understanding of midwifery care philosophy “women-centred care”**

**Topics**	**Construction of questions**	**Number of true-false questions**
Personalized care	- Students’ experience gained during clinical practice concerning their understanding of women’s needs.	5
	- Students’ comprehension about the difference of each woman’s needs that students give a midwifery care.	
	- Students’ experience about offering a helping hand to the woman who has a special need.	
	- Students’ experience how to recognise every woman’s right to self‑determination in attaining choice of care for woman herself.	
Holistic care	Students’ understanding concerning a holistic approach and recognition to each woman’s social, emotional, physical, spiritual and cultural needs, expectations and context as defined by the woman herself	3
Partnership care	- Students’ mean of partnership with women to give the necessary support, care and advice during pregnancy, labour and the postpartum period.	4
	- Students’ understanding how the midwives provide women with appropriate information and advice in a way that promotes participation and facilitates informed decision making.	
Collaborative care	- Students’ understanding how the midwives take a dicision to referral in a timely manner when problems arise on their client during pregnancy, birth, and the postpartum period.	3
	- Students’ understanding on how the midwife is expected to continue providing supportive care after transfer and be going to resume primary care if appropriate.	
Evident-based care	Students’ ability to inform and give midwifery care based on scientific evidence that they know.	3
Total		18

The development of the questionnaire was done using references from the International Confederation of Midwives (ICM) [1,6]. The five aspects of women-centred care philosopy was than adapted into 18 questionnaire items by the research team, followed by consultation on the appropriateness, relevance and readability of the questionnaire to the professional groups relevant to midwifery education (18 Institutional Lecturers, 13 Clinical Midwives, and 2 experts from Indonesia Midwifery Association) by two times focus group discussion.

The questionnaire was administered to both groups in their first week of the programme and six months after completing the study. The Cronbach’ alpha for the questionnaires was 0.754, meaning that all items are reliable [25]. Pre-test questionnaire and consent forms were distributed to students one week prior to the commencement of the study. Students were informed about the voluntary nature of the study and assured of confidentiality and anonymity. To enable matching of pre- and post-test data, students were asked to generate their own identification code using a combination of their birthday initials. Students completed and returned the questionnaire during the session. A second questionnaire was given to students when they returned to the school following their clinical experience. Again, the questionnaire was distributed and collected during a lecture held at the school. All students completed both the pre-test and post-test surveys. This was a 100% response rate. The quantitative data were analysed using SPSS, and the independent sample t-test was conducted.

### Ethical considerations

The study was conducted after approval had been obtained from Gadjah Mada University Human Research Ethics Committee (Ref: KE/FK/812/EC). In addition, permission to conduct the study was obtained from Director of the School of Midwifery. All participants were informed of the objective and design of the study and a written consent received from the participants to participate on the study. Staff members who did not have a direct power relationship with the student on their course took consent. Prior to clinical placement, consent was obtained from patients willing to participate in the study.

## Results

### Students’ understanding of midwifery care philosopy

Table 2 shows the results of students’ understanding between the experiment group and the control group. There was no significant difference in students’ understanding of midwifery care philosopy between the two groups before the study (P > 0.05), but a significant difference occurred after the study (P < 0.01).

**Table 2:** **Means for students’ ratings on five aspects of midwifery care philosopy**

**Aspects**	**(n = 54) Experiment group (CoC)**	**(n = 52) Control group (Fragmented)**	**t**	**Significant between subterm**	**Significant between groups**
***Pre-clinical***					
Personalized care	1.39 (.712)	1.42 (.723)	-0.245	0,087	
Holistic care	2.59 (1.055)	2.65 (1.136)	-0.288	0,774	
Partnership care	1.04 (.672)	1.04 (.685)	-0.011	0,650	**P>0.05**
Collaborative care	2.17 (.746)	2.23 (.703)	-0.455	0,991	
Evidence-based care	1.17 (.607)	1.15 (.607)	0.109	0,914	
**Total**	**8.35 (1.824)**	**8.50 (1.698)**	**-.432**	**0,666**	
***Post-clinical***					
Personalized care	2.63 (.487)	1.67 (.648)	8.606	0,000	
Holistic care	4.46 (.539)	3.37 (.886)	7.734	0,000	
Partnership care	2.65 (.482)	1.38 (.491)	13.365	0,000	**P<0.01**
Collaborative care	3.57 (.499)	2.77 (.614)	7.418	0,000	
Evidence-based care	2.65 (.482)	1.46 (.576)	11.519	0,000	
**Total**	**15.96 (1.063)**	**10.65 (1.170)**	**24.469**	**0,000**	
**Significant between subterm within the same group**					
Personalized care	0,000	0,000			
Holistic care	0,000	0,000			
Partnership care	0,000	0,000			
Collaborative care	0,000	0,000			
Evidence-based care	0,000	0,000			
**Significant pre and post intervention**	**P < 0.01**	**P < 0.01**			

Independent t-test (df = 104 ; CI 95%) t table = 1.65964.

In relation to the main impacts for models, students’ ratings on all five aspects were significantly different between the groups on the post-clinical survey: personalized care (t = 8.606, p < .01; holistic care (t = 7.734, p < .01; partnership care (t = 13.365, p < .01; collaborative care (t = 7.418, p < .01; and evidence based care (t = 11.519, p < .01). The mean score for students with the CoC clinical learning model on all five aspects (15,96) was higher than that for the students with the fragmented care model (10.65).

## Discussion

We found that the CoC clinical learning model increased students’ understanding of midwifery care philosopy to a higher level compared to the fragmented care model. This finding suggests that students who use a CoC clinical learning model understand how to give better midwifery care based on the midwifery care philosophy during their clinical practices. Learning through relational continuity of care is important for promoting personal growth for the midwifery students and therefore they are in a better position to offer holistic care. Students in the CoC learning groups showed more understanding about midwifery care philosophy than those in the fragmented care learning model. Continuity of care in the relationship with women and the practices of holistic midwifery care seems to be more satisfying for students, and enhances their self-confidence as midwives [14].

In this study we also found that students in both groups were significantly better in understanding the five aspects of midwifery care philosophy (Table 2). However, students in the CoC learning groups had a significantly better understanding than the control group. Therefore, the CoC learning model provides a more meaningful educational impact than the fragmented learning model. Possibilities for this include the more intensive and longer relationships between the students and the women during all phases of pregnancy, labor and post delivery, and/or the improvement in comprehension of the midwifery care philosophy into practice.

Previous studies have shown that students identified the importance of providing care and support in a meaningful and woman-focused manner from early pregnancy throughout the childbearing period, and explained that this was a valuable learning experience [16,20,27,28]. A study by Seibold [22] reported that students experienced a personal transformation and rated their follow up experience as highly valuable. This study found that providing this experience to all midwifery students enabled them to better understand all aspects of midwifery care philosophy,

Our study showed that a CoC clinical learning model provided opportunities for the students to practise women-centred care. It was the students’ presence and focus upon women which empowered the women in this study.

In relation to changes following the clinical experience, all students who implemented the CoC learning model felt more competent and satisfied. This indicated that the students developed their skills and care practices during the experience and consequently were satisfied with the practicum. The importance of clinical experience for competence and skill development has been reported in the literature [22]; our study confirms the importance of clinical experience for ongoing student development. It is interesting to note that the competence and satisfaction of students using the CoC learning model improved in this study. The results suggest that clinically following women during pregnancy, childbirth and post partum enabled students to gain appropriate and satisfiying learning experiences. This study indicates that there should be no hesitation in adopting a CoC clinical learning model to enable midwifery students to develop relevant skills and competence.

This study also confirmed the elements of midwifery care philosophy that contributed most to a positive learning experiences for students. Consistent with other research [14,16,22,28,29], these elements were related to supporting students obtain their learning, by being a part of a midwifery care team, and thus feeling valued. The findings strongly suggested which aspects of health care agencies fostered not only the development of students’ confidence and competencies, but also students’ understanding of midwifery care philosophy. It is vital that stakeholders in clinical education ensure that the clinical learning model not only recognise these issues but also have the relevant structures in place to support learning. This should include providing diverse experiences and recognizing students as valued members of the health care team.

There are many benefits associated with the CoC learning model in midwifery care [30]. The CoC learning model in midwifery care is based on the premise that pregnancy and birth are normal, women-centered life events. It is assumed that the underpinning philosophy of midwifery care is based upon the natural ability of women to experience birth with minimum or no routine intervention. The CoC model of care offers greater relationship continuity by ensuring that childbearing women receive their ante-, intra- and postnatal care from one midwife or her/his practice partner. In our study, the CoC learning model that was offered to the students gave additional benefits, such as early detection and prompt treatment for high risk pregnancies. Of the 108 women who participated in this study, there was zero maternal mortality at the end of the CoC learning model implementation. Therefore, through the CoC model of care that is implemented in midwifery care system, it is in line with targets 4 & 5 of MDGs to reduce maternal and infant mortality.

This study has also been producing a 6 month CoC learning module for a three year midwifery education programe. The CoC experience is designed for midwifery students to be aligned with women, so that they are embedded in the practices in the community and daily service provision. Furthermore, the CoC experience is intended to provide midwives who adopt continuity of care and a women-centred care philosophy as their ideal for future professional practice. However, as previously studies in our study identified, continued exposure to midwifery practices offers great benefits to students in developing their own personal midwifery identity and philosophy.

### Study limitations and recommendations

While this study reported that the CoC learning model resulted in many educational benefits, student accessibility issues such as cost, transportation and accommodation are important aspects that should be taken into account. This suggests that certain background factors must be addressed in order for students to consider a CoC clinical placement.

Further study should address concurrent improvement in care practices or other relevant skills in the CoC clinical experience. We suggest that longer periods of clinical practice may be required with the CoC learning model to optimize student opportunities for developing relevant skills and competence.

Although only the students’ understanding of midwifery care philosophy was analyzed in this study, the findings demonstrated that further research should be continued to understand student competence and skills. This will further strengthen the midwifery curriculum and contribute positively to the women-centred care that underlies midwifery practices.

## Conclusion

The CoC learning program provides a unique learning opportunity for students to understand what the CoC as part of midwifery care philosophy offers to women. Being aligned with women and developing effective relationships with them enable students to have a unique view of midwifery practice which instills a strong philosophy of women-centred care.

## Additional file

Additional file 1:
**Modules of Continuity of Care Learning Model.** For a 6 month Clinical Placement In Indonesia Midwifery Education Programe.

## Abbreviations

CoC: Continuity of care
ICM: International confederation of midwives
MDGs: Millenium development goals
