# Supplementary material for: Students’ understanding of “Women-Centred Care Philosophy” in midwifery care through Continuity of Care (CoC) learning model: a quasi-experimental study
Source: BMC Nurs. 2015 Apr 22;14:22. doi: 10.1186/s12912-015-0072-z (PMC4416326; doi:10.1186/s12912-015-0072-z)
Supplement: Additional file 1: — Modules of Continuity of Care Learning Model. For a 6 month Clinical Placement In Indonesia Midwifery Education Programe. [file 12912_2015_72_MOESM1_ESM.doc]

**Modules of Continuity of Care Learning Model**

**For a 6 month Clinical Placement In Indonesia Midwifery Education Programe**

**Rationale:**

This module will critically analyse the concepts in women-centred care such as choice control and empowerment. These will provide students with an insight into the role of a midwife in normal midwifery practices. The students will gain experience which they have been involved and relate it to an aspect of midwifery care.

**Module Aim:**

This module will further develop through analysis and reflection of the students’ personal and professional knowledge and skills in the provision of midwifery care.

**Procedures:**

1. **Women recruitment**


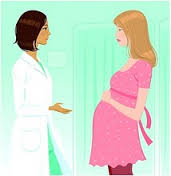


- Students are required to

recruit and manage their

own caseload of women

for these 2-3 CoC

experiences.

- Student initiation of contact with women/ family in the community clinic or home.
- The recruitment process is assisted by a clinical midwife, that will become the students’ partner with during clinical practice.

1. **Antenatal care by midwife student**


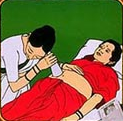


- Students will enhance

existing clinical skills

in the initial and sub-

sequent assessment,

examination and care

of the mother and fetus

during pregnancy, both normal and abnormal.

- Students will have the opportunity to provide antenatal care at privately practicing, midwife’s clinics, in the

This module was adopted from Yanti, et al. (2014)

woman’s own house and at the hospital if it is required**.**

- Students discuss each case with their responsible clinical midwife to further provide an opportunity for learning on antenatal.
- Students should write a portofolio and logbook in which they can document their progress and achievement for practice outcomes of antenatal care.

1. **Tri-partite meetings I**

- It is done before the women on their date of labour that students are following the CoC.
- These meetings provide a forum for students, clinical midwife, and the supervising midwife teacher to discuss any issues or concerns that might have arisen during antenatal.
- These provide an opportunity for feedback and professional discourse and focus on issues of accountability, record keeping and communication among parties.

1. **Intranatal care by student midwife**


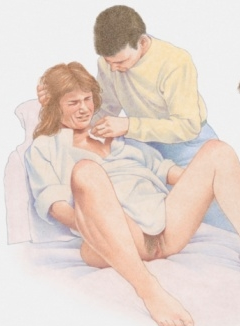

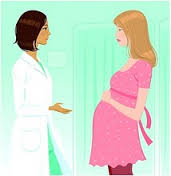

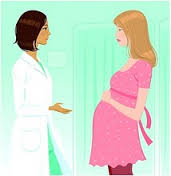


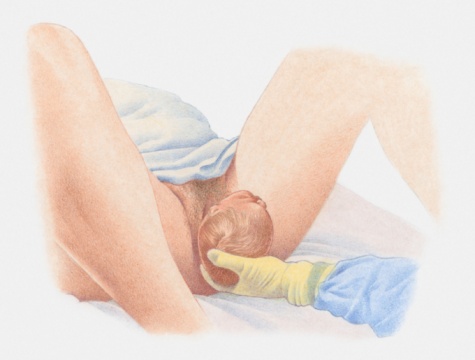


- In this stage, students will gain further experience and skill in caring during labour and birth for the same women that they follow from antenatal assisted by the responsible midwive.
- Students will be expected to conduct

normal deliveries, gain experience in repairing the perineum and care for high dependency women using evidence

based practice, during intranatal experience.

1. **Tri-partite meetings II**

- It is done during 24 hours after birth.
- Any issues or concerns that might have arisen during intranatal are discussed at these moments.

1. **Postnatal care by midwife student**


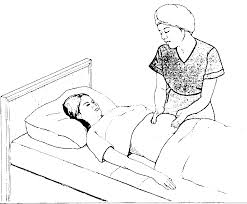


- Students will experience

the role of conduct of the

midwife in the management

of the postnatal period for the

women who recieve care

from the students prevously.

- These experiences are

encompassing in the home, transfer and discharge procedures to include: hospital and community liaison, community services and follow up care, records and documentation, participate in the completion of statutory records and notification, recognise early signs of deviation from normal and referral to relevant specialist.

1. **Tri-partite meetings III**

- It is done 2 weeks after birth.
- These meetings are encompassing any issues or concerns that might have arisen during postnatal and the previous periods (antenatal and intranatal) are discussed at these times, that provide an pportunity for feedback among parties.

**Necessary notes:**

- The overall length of the clinical placement is 6 months including holidays.
- To facilitate subjective monitoring and self assessment students will compile a portfolio of clinical experience in each phase.
- Students will be asked to use their portfolio to demonstrate to the mentor midwife and the personal tutor how they have achieved the learning outcomes of the module.
- Students will continuously assessed by their mentor midwife.

**References:**

1. Bennett R Brown LK (1999) Myles Textbook for Midwives 13^th^ ed Edinburgh Churchill Livingstone.
2. Sweet BR (Ed) (1997) Mayes’ Midwifery: A Textbook for Midwives 12^th^ ed London Bailliere Tindall.
3. NMC REVIEW (2007) : BSC In Midwifery Studies 18-Month Programme. Queen’s University Belfast.
4. Glover, P. Follow through experiences - as midwifery curriculum. Australian Midwifery Journal 2003, 16 (2):, 5-6.
5. Fry J, Rawson S, Lewis P. Student caseloading: preparing and supporting students. British Journal of Midwifery 2008, 16(9): 568–73.
6. Giarratano G: Women-centred maternity nursing education and practice. J Perinat Educ 2003, 12(1):18–28.
7. Homer, C., Brodie, P., Leap, N. (Eds.). Midwifery Continuity of Care: A Practical Guide. Elsevier, Sydney; 2008.
